# Statins Decrease Neuroinflammation and Prevent Cognitive Impairment after Cerebral Malaria

**DOI:** 10.1371/journal.ppat.1003099

**Published:** 2012-12-27

**Authors:** Patricia A. Reis, Vanessa Estato, Tathiany I. da Silva, Joana C. d'Avila, Luciana D. Siqueira, Edson F. Assis, Patricia T. Bozza, Fernando A. Bozza, Eduardo V. Tibiriça, Guy A. Zimmerman, Hugo C. Castro-Faria-Neto

**Affiliations:** 1 Laboratório de Imunofarmacologia, Instituto Oswaldo Cruz, Fundação Oswaldo Cruz, Rio de Janeiro, RJ, Brazil; 2 Laboratório de Investigação Cardiovascular, Instituto Oswaldo Cruz, Fundação Oswaldo Cruz, Rio de Janeiro, RJ, Brazil; 3 Instituto de Pesquisa Clínicas Evandro Chagas, Fundação Oswaldo Cruz, Rio de Janeiro, RJ, Brazil; 4 Department of Internal Medicine, University of Utah School of Medicine, Salt Lake City, Utah, United States of America; London School of Hygiene and Tropical Medicine, United Kingdom

## Abstract

Cerebral malaria (CM) is the most severe manifestation of *Plasmodium falciparum* infection in children and non-immune adults. Previous work has documented a persistent cognitive impairment in children who survive an episode of CM that is mimicked in animal models of the disease. Potential therapeutic interventions for this complication have not been investigated, and are urgently needed. HMG-CoA reductase inhibitors (statins) are widely prescribed for cardiovascular diseases. In addition to their effects on the inhibition of cholesterol synthesis, statins have pleiotropic immunomodulatory activities. Here we tested if statins would prevent cognitive impairment in a murine model of cerebral malaria. Six days after infection with *Plasmodium berghei* ANKA (PbA) mice displayed clear signs of CM and were treated with chloroquine, or chloroquine and lovastatin. Intravital examination of pial vessels of infected animals demonstrated a decrease in functional capillary density and an increase in rolling and adhesion of leukocytes to inflamed endothelium that were reversed by treatment with lovastatin. In addition, oedema, ICAM-1, and CD11b mRNA levels were reduced in lovastatin-treated PbA-infected mice brains. Moreover, HMOX-1 mRNA levels are enhanced in lovastatin-treated healthy and infected brains. Oxidative stress and key inflammatory chemokines and cytokines were reduced to non-infected control levels in animals treated with lovastatin. Fifteen days post-infection cognitive dysfunction was detected by a battery of cognition tests in animals rescued from CM by chloroquine treatment. In contrast, it was absent in animals treated with lovastatin and chloroquine. The outcome was similar in experimental bacterial sepsis, suggesting that statins have neuroprotective effects in severe infectious syndromes in addition to CM. Statin treatment prevents neuroinflammation and blood brain barrier dysfunction in experimental CM and related conditions that are associated with cognitive sequelae, and may be a valuable adjuvant therapeutic agent for prevention of cognitive impairment in patients surviving an episode of CM.

## Author Summary

Cerebral malaria (CM) is the direst consequence of *Plasmodium falciparum* infection. Cognitive impairment is a common sequela in children surviving CM. Identification of adjunctive therapies that reduce the complications of CM in survivors is a priority. Statins have been suggested for the treatment of neuroinflammatory disorders due to their pleiotropic effects. Here, we examined the effects of lovastatin on neuroinflammation in experimental CM, and its effect on the prevention of cognitive impairment. Lovastatin reduced adhesion and rolling of leukocytes in brain vessels, inhibited blood-brain barrier disruption, and reversed decreases in cerebral capillary density. Lovastatin also inhibited ICAM-1 and CD11b mRNA expression while increasing HMOX-1 mRNA levels. Proinflammatory cytokines and markers of oxidative stress were lower in the brains of infected mice treated with lovastatin. Lovastatin administered together with antimalarial drugs during the acute phase of the disease-protected survivors from impairment in both contextual and aversive memory 15 days after infection. Similar results were observed in a model of bacterial sepsis. Our findings support the possibility that statins may be valuable pharmacologic tools in treatment of patients with neuroinflammation associated with severe systemic inflammatory syndromes. Clinical trials with statins in CM and sepsis should be speedily considered to examine this point.

## Introduction

The burden of malaria is enormous with more than 40% of the world's population at risk for infections caused by *Plasmodium* parasites [1]. *P. falciparum* is the principal cause of syndromes of severe malaria, including cerebral malaria, which involves neurologic and systemic manifestations and in which coma is the defining feature [2]. While only about 1% of *P. falciparum* infections progress to cerebral malaria, mortality is around 10–20% of affected patients [3]. Prevention of plasmodial infection, development of new anti-malarial drugs with consistent efficacy, and identification of adjunctive therapies that can reduce organ damage in cerebral malaria and other forms of severe malaria are public health and scientific objectives of highest priority [1], [4].

Children in the sub-Saharan Africa are disproportionately at risk for developing cerebral malaria [3] and there is now evidence that many patients who are effectively treated and survive cerebral malaria have long term physical, neurologic and neurocognitive dysfunctions and thus present a second clinical challenge [3], [5]–[7]. Approximately 500,000–800,000 children develop cerebral malaria each year in sub-Saharan Africa alone, suggesting that persistent cognitive dysfunction occurs in a significant number of young survivors. In fact, neurocognitive impairments were detected in as many as 26% of African children when follow up testing was done 6 months to 9 years after treatment of acute cerebral malaria [6]–[8]. The potential impact on learning and behavioral function and its societal consequences is staggering.

We recently reported studies employing mouse models of cerebral malaria with persistent cognitive dysfunction after rescue therapy with conventional anti-malaria drugs [9]. Cognitive impairment in contextual and aversive memory occurred in mice of susceptible genetic backgrounds infected with *Plasmodium berghei* ANKA (PbA) despite rescue therapy that was given early, when neurologic manifestations were relatively subtle and before the onset of coma, seizures, and other manifestations [9]. These models may provide important insights into the pathogenesis of cognitive dysfunction associated with cerebral malaria and related disorders that may be relevant to human conditions [7]. While differences between murine models of CM and the human syndrome are often emphasized [10], [11], there are also important similarities [3], [7], [12]–[14]. Nevertheless, caution must be exerted when translating experimental findings to the clinical scenario.

Exacerbated inflammatory responses are major causes of organ dysfunction during severe infectious syndromes. In cerebral complications of systemic infections, pro-inflammatory cytokines and chemokines activate leukocytes and endothelial cells and lead to microvascular plugging, impaired blood flow, breakdown of the blood-brain barrier (BBB), local neuroinflammation, and apoptosis. All of these are observed, for example, in animal models of sepsis and malaria [9], [15], [16]. In addition to directly injuring neurons, neuroinflammation activates quiescent microglia, which are resident cerebral macrophages that can play a pivotal role in brain damage from systemic inflammation [17].

Mechanisms that underlie cognitive dysfunction after brain involvement in severe infectious syndromes and measures that can be used to prevent and treat it are, however, largely unexplored. We propose that drugs with pleiotropic effects on inflammation and metabolism could be ideal candidates to target neuroinflammation and decrease cognitive impairment after severe infectious syndromes. Existing data indicate that statins may be such agents and that they can modify two processes leading to brain injury: neuroinflammation and activation of proinflammatory microglia [18], [19]. Statins, therefore, may counteract the pro-inflammatory phenotype and microglial activation during critical illness. Their actions favor an antiinflammatory switch that may contribute to neurovascular stabilization and neuronal healing rather than damage. In this study we present the first evidence that adjunctive treatment with statins prevents cognitive impairment after cerebral malaria. We also provide evidence that statins prevent cognitive dysfunction in experimental sepsis. The effects of statins in these models are consistent with activities that decrease inflammation and microglial activation and improve microvascular parameters in the brain. These observations have clear translational implications, and suggest that statins should be considered key candidates for clinical trials aimed at cognitive sequelae in patients with cerebral malaria and other severe infectious syndromes.

## Results

### Cognitive impairment in animals rescued from CM by antiplasmodial drug treatment is abrogated by adjuvant lovastatin administration

**Figure 1:**
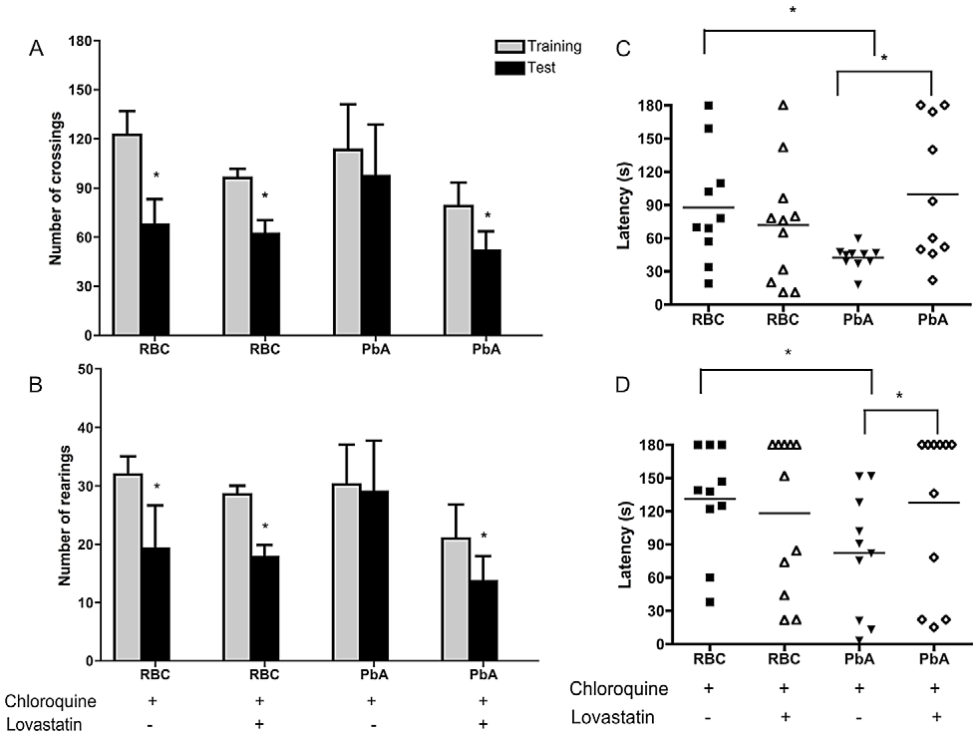
Lovastatin treatment prevents contextual and aversive memory impairment after CM. C57BL/6 mice (n = 12–20/group) were infected with PbA (10^6^ PRBC). As a control, one group was inoculated with the same number of uninfected RBC (n = 6–12/group). Starting on day 6-post infection, uninfected and PbA-infected mice were divided into 2 groups and treated orally with chloroquine (25 mg/kg b.w.), or with the combination of chloroquine/lovastatin for 7 days. On days 15 and 16 post-infection all the animals were submitted to open field training and test sessions (A–B), and to inhibitory avoidance sessions tasks (C–D). Data are expressed as mean ± SEM of crossings (A) and rearings (B) in training (*gray bars*) and test (*black bars*) sessions. *Significant difference between groups in training and test sessions (p<0.05, Student's t test). C–D: animals were subjected to a training session in which the latency time on the platform is recorded when an electrical shock is given immediately after the mice step down onto the bars. (C) 1.5 (Short-term memory) and (D) 24 h (long-term memory) aversive memory was tested by recording the latency time on the platform (with a cut-off of 180 sec). Data are expressed as individual values and horizontal lines represent the mean of latency, in seconds; *Significant difference compared with uninfected controls and between infected groups (comparisons among groups were performed by Mann-Whitney U test, *p<0.05*).

**Figure 2:**
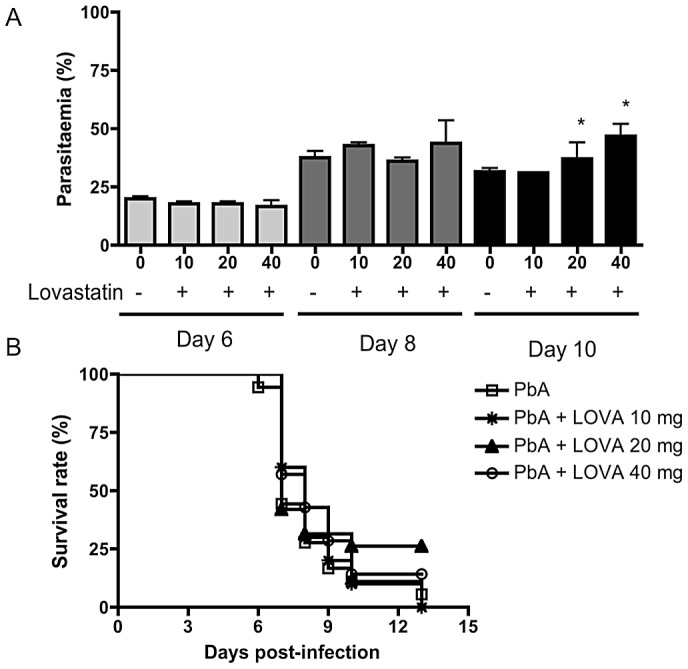
Effect of lovastatin on parasitemia and survival rate after infection with PbA (n = 12–20/group). Data on parasitemia (A) are shown as mean ± SEM. On day 6 post-infection mice were separated in two groups and one received lovastatin (10–40 mg/kg b.w.) daily for 7 days. Comparisons of C57BL6-PbA versus C57BL6-PbA treated animals (*) were significant by Mann-Withney Comparison test. There was no difference in parasitaemia when PbA and PbA-lovastatin treatment were compared (Mann-Withney Comparison test). (**B**) No differences in survival were observed among the groups (Log-rank (Mantel-Cox) and Gehan-Breslow-Wilcoxon tests).

Long-term cognitive impairment was described in African children surviving CM [6], [8], and we recently found that cognitive impairment is mimicked in experimental models of CM [9]. Here, we show that oral lovastatin administered concomitantly with chloroquine prevented cognitive impairment detected 15 days after CM (Figure 1). Chloroquine and lovastatin treatment were started at day 6-post infection and continued for 7 days in animals that developed early CM based on positive scoring for clinical signs in a complex battery of physiologic tests [9]. Lovastatin (10–40 mg/kg) did not have anti-parasitic effects alone (Figure 2A) or interfere with the anti-parasitic effect of chloroquine, since parasitemia levels were similar in animals receiving chloroquine or the combination of drugs (not shown). Further, lovastatin treatment did not prevent mortality when given alone despite the slight delay effect observed in Figure 2B.

At day 15 animals receiving the combination therapy or chloroquine alone had no detectable parasitemia or any clinical signs of disease (not shown) and were, therefore, subjected to cognitive evaluation tasks. We observed that PbA-infected mice that were positive for clinical signs of CM on day 6 after infection and were rescued by chloroquine treatment lacked contextual memory. This was demonstrated by habituation to an open-field [9] since the animals showed no significant differences in the number of crossings and rearings when training and test sessions were compared (Figure 1 A, B). In contrast, animals that were treated with lovastatin in addition to chloroquine showed a significant reduction in the number of crossings and rearings when comparing test and training sessions (Figure 1A, B), indicating intact contextual memory in those mice rescued from CM and treated with lovastatin. In a parallel set of experiments, animals were evaluated for aversive memory integrity by a step-down inhibitory avoidance test [9]. Both short and long-term memory during the avoidance task were affected in mice rescued from CM by chloroquine since the times of latency on the platform in those animals were significantly shorter then in uninfected controls (Figure 1C and D, respectively). Lovastatin treatment also prevented loss of aversive memory in mice receiving the combination therapy, as indicated by longer times of latency on the platform when compared to animals treated with chloroquine alone (Figure 1C, D).

**Figure 3:**
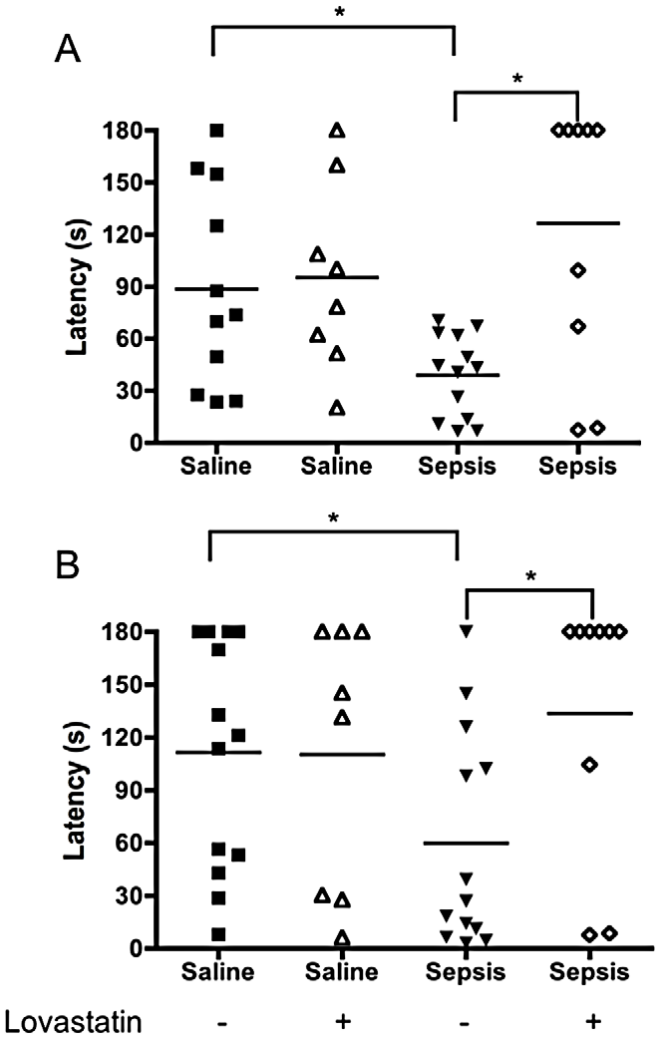
Lovastatin treatment prevents impairment of short and long-term aversive memory after sepsis. C57BL/6 mice (n = 5–10/group) were inoculated with fecal supernatant (2.5 mg/g b.w.). For control, one group was inoculated with saline (0.9%). Control and mice challenged with fecal supernatant were divided and one group was treated with lovastatin (20 mg/kg b.w.) 1 hour prior to feces or saline injection respectively, and every 24 h for 3 days. All the animals received imipenem (10 mg/kg b.w.) 6 hours after inoculation and every 24 h for 3 days. On day 15 all animals were subjected to a training session of inhibitory avoidance task, where the latency time on the platform is recorded and an electrical shock is given immediately after the mice step down onto the bars. (A) 1.5 (Short-term memory) and (B) 24 h (long-term memory) aversive memory was then tested by recording the latency time on the platform (with a cut-off of 180 sec). Data are expressed as individual values and horizontal lines represent the mean of latency, in seconds; significant difference compared between saline versus feces injected mice (comparisons among groups were performed by Mann-Whitney U test, *p<0.05).

Interestingly, lovastatin treatment also prevented cognitive impairment after bacterial sepsis (Figure 3). We found that animals rescued by antibiotic therapy and fluid resuscitation had significant cognitive impairment detected both by the habituation to the open field test (data not shown) and by the step-down inhibitory avoidance test performed 15 days after the infection. Adjuvant treatment with lovastatin together with antibiotics prevented the cognitive impairment (Figure 3), in a fashion similar to that we observed in the malaria model (Figure 1). Cognitive damage after severe experimental sepsis was previously shown to be reversible by treatment with anti-oxidant drugs [20].

### Lovastatin treatment increases functional capillary density and decreases leukocyte-endothelial interactions in a model of CM

**Figure 4:**
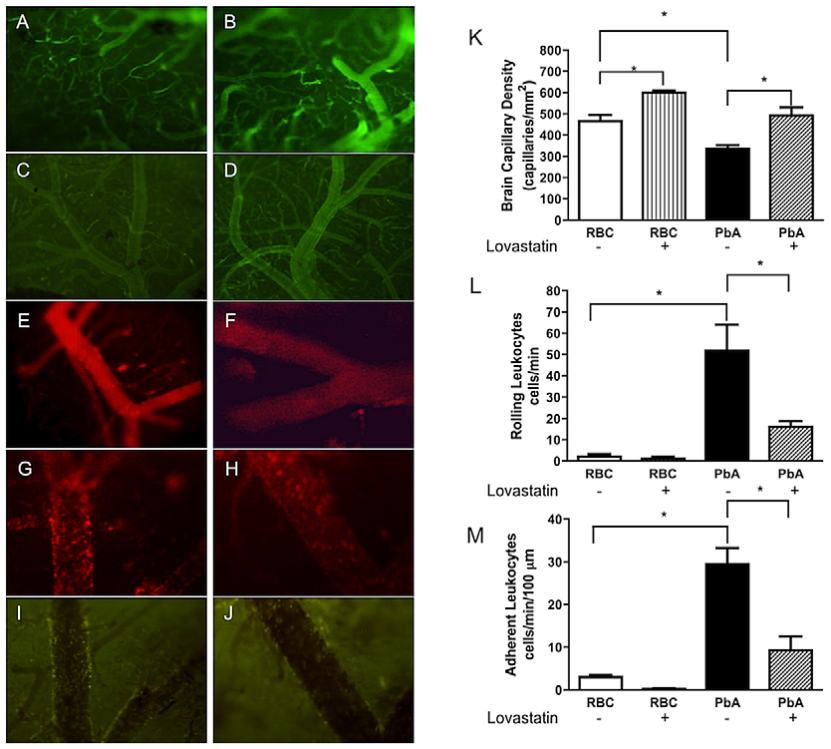
Lovastatin improves microvascular function and decreases leukocyte rolling and adhesion during PbA infection. A–J: representative images of fluorescence intravital microscopy of pial vessels of uninfected mice (A and B; Magnification 100×) or infected mice (C and D) treated with vehicle or lovastatin, respectively (Magnification 40×). Venules with adherent and rolling rhodamine-labeled leukocytes in uninfected mice (E and F) or infected mice (G and H) treated with vehicle or lovastatin, respectively (Magnification 200×). Rhodamine-labeled leukocytes were associated with the venular endothelium in animals treated with vehicle (G) whereas they were largely free in the blood stream in lovastatin-treated mice (H). Fluorescent *Plasmodium berghei* (GFP)-infected RBC in venules of mice treated with vehicle (I) or lovastatin (J) (Magnification 200×). Mean ± SEM of functional capillary density (K), of rolling-rhodamine labeled leukocytes (L), and of number of adherent leukocytes (M) in pial venules (n = 6/group); *p<0.05 in relation to control (RBC) and vehicle-treated groups (Bonferroni's Multiple Comparison Test) and between PbA and PbA-lovastatin group (Bonferroni's Multiple Comparison Test; Scale bar, 100 µm).

In order to investigate potential mechanisms of the neuroprotective effect of statins in experimental CM we analyzed the influence of lovastatin on neuroinflammatory parameters after the initial signs of CM. Six days after the infection of C57Bl/6 mice with PbA, the animals demonstrated clear signs of CM (which were not present in controls injected with uninfected RBC), as detected by a modified SHIRPA protocol [9] (zero score points in controls versus 5.7±1.7 in PbA-infected mice, p<0.05). Mice that scored above 3 points, and were therefore considered positive for CM, were then taken to intravital microscopic examination of the pial microvessels through a cranial window. As illustrated in Figure 4, there was a marked decrease in functional capillary density (PbA-infected 336.7±15.6 versus control 4656±29.1 capillaries/mm^2^, p<0.05). In parallel, there was an increase in rolling (PbA-infected 51.8±12.2 versus control 2.2±1.1 cells/min; p<0.05) and adhesion (PbA-infected 29.5±3.7 versus control 2.2±1.0 cells/min; p<0.05) of leukocytes in brain venules when infected and uninfected animals were compared. The decrease in capillary density was reversed in infected animals that were treated with lovastatin (Figure 4A–D, K). In addition, lovastatin also reduced leukocyte rolling and adhesion (Figure 4E–H, L, M). Furthermore, the accumulation of infected RBC in brain vessels identified by GFP-labeled PbA was reduced in lovastatin-treated animals (Figure 4I, J).

### Lovastatin protects against blood-brain barrier disruption during experimental CM

**Figure 5:**
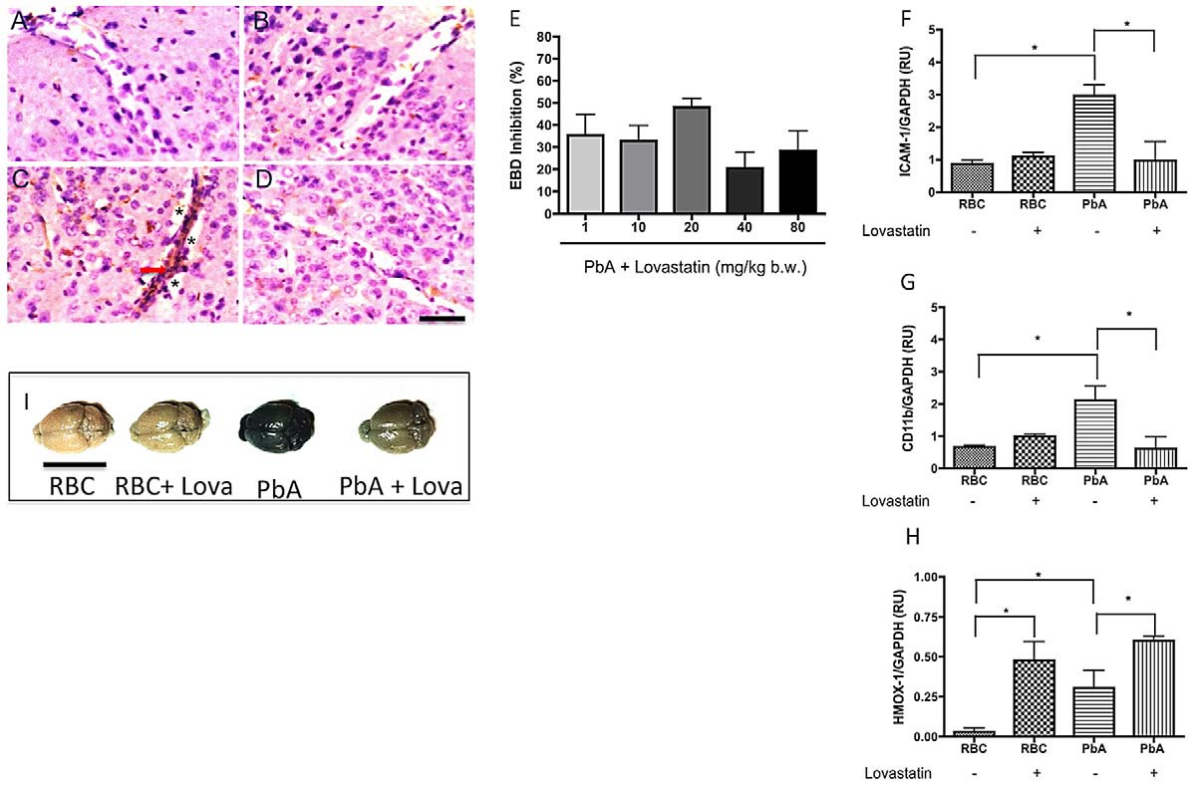
Lovastatin treatment decreases ICAM-1 and CD11b expression and vascular permeability and induce HMOX-1 expression in the brains of mice with CM. Panels illustrate histological examinations of cerebral cortex on day 6 post-infection, immunostained for ICAM-1 (brown and arrow) and counterstained with hematoxylin-eosin. Brain histology was examined using tissue from the following groups of animals: uninfected (A), uninfected treated with lovastatin (B), PbA-infected (C), and PbA-infected treated with lovastatin (D). Vascular congestion and edema (*) were observed in all PbA-infected mice, but were not seen in controls or treated animals. Scale bar: 50 µm. E: Dose-response relationships for effects of lovastatin on edema formation measured by Evans Blue Dye accumulation in the brain tissue on day 7 post-infection. F–H: ICAM-1, CD11b, and HMOX-1 expression in brain samples evaluated by semi-quantitative PCR on day 6 post-infection; *p<0.05 by Tukey's Multiple Comparison Test (n = 3–5/group). I-representative macroscopic image of Evans blue dye extravasation in the brain, demonstrating a marked reduction in PbA mice treated with lovastatin (20 mg/kg) on day 7-post infection.

BBB disruption is a common consequence of human and experimental CM [21]–[23]. Using Evans blue dye as a marker for increased vascular permeability we observed that PBA-infected mice that were positive for clinical signs of CM on day 6 after infection had a marked increase in BBB permeability (an average increase of 211% in Evans blue dye extravasation as compared to uninfected control – from 5.7 µg/mg of brain tissue in uninfected controls to 17.7 µg/mg of brain tissue in PbA infected mice). Animals begun on lovastatin treatment (1–80 mg/kg) on day 6 and examined one day later, however, showed a significant decrease in BBB disruption (Figure 5E). The beneficial effect was more pronounced with 20 mg/kg (around 48% of inhibition - Figure 5E, I). These findings were confirmed by histological analysis, which demonstrated reduced perivascular edema and congestion on day 6-post infection in lovastatin-treated animals (Figure 5A–D), indicating that BBB disruption can be prevented, or at least lessened, by statin treatment in this experimental model. In addition, ICAM-1 expression was decreased in the brain microvessels in animals started on lovastatin treatment one day before (Figure 5A–D and F), consistent with decreased adhesion of leukocytes in mice treated with lovastatin (Figure 4). Furthermore, treatment with lovastatin at day 6 post-infection also decreased CD11b expression (Figure 5G), potentially contributing to decreased accumulation of leukocytes in inflamed vessels or cerebral tissue [24] (Figure 4G–J and 4L–M).

Hsu and coworkers [25] showed that the treatment with statins increased heme-oxygenase 1 (HMOX-1) expression in brain, lung, heart and liver of healthy mice. Our findings (Figure 5H) show that the treatment of mice with lovastatin enhances the expression of HMOX-1 both in healthy and infected brains, as shown by PCR (Figure 5H) and Western blotting (data not shown).

### Lovastatin treatment reduces cytokine levels in the brains of PbA-infected mice

**Figure 6:**
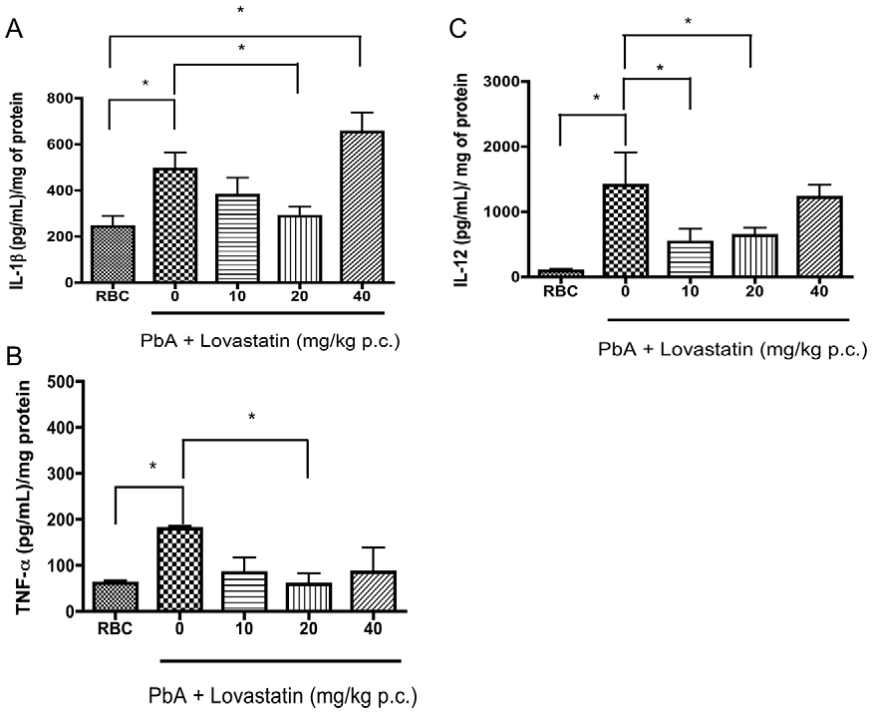
Lovastatin treatment reduces pro-inflammatory cytokine levels in the brains of animals with CM. IL-1β (A), TNF-α (B), IL-12 (C) levels were determined by ELISA in brains of mice 6 days after infection with PbA (10^6^ PRBC, n = 6–10/group). Lovastatin (10–40 mg/kg) was orally administered on day 6 post-infection together with chloroquine (25 mg/kg). *p<0.05 or less (Tukey's Multiple Comparison test) in relation to non-infected group and #p<0.05 or less in relation to PbA-infected group (Mann-Withney U Test).

Anti-inflammatory effects of statins have recently been described in several different models and conditions [26], [27]. One of the mechanisms is the modification of the pattern of cytokines/chemokine that are expressed [28]. We, therefore, investigated the ability of lovastatin to modify cytokine and chemokine responses in this model of CM. We found that IL-1β, TNF-α and IL-12 levels were elevated in the brains of mice on day 6-post infection, and were reduced by lovastatin treatment (Figure 6). MCP-1 levels were also decreased by lovastatin (20 mg/kg) from 2074±571.1 in untreated mice to 1212±946.2 pg/mL in animals receiving lovastatin. Interestingly, RANTES levels were not altered in infected animals nor were they affected by lovastatin treatment (supplemental data 1). We also observed high levels of TNF-α and MCP-1 in the plasma on day 6-post infection (TNF: 106.1±22.1 pg/mL in uninfected, 855.9±262.6 pg/mL in PbA-infected animals; MCP-1, 342.2±100.2 pg/mL in uninfected, 1189±275.6 pg/mL in PbA-infected animals; p<0.05 by Mann-Withney test in each case). Treatment with lovastatin reduced plasma levels of both TNF-α (855.9±262.6 pg/mL in saline-treated, 456.9±66.8 pg/mL in lovastatin-treated animals, p<0.05 by Mann-Withney test) and MCP-1 (1189±275.6 pg/mL in saline-treated, 602.5±179.9 pg/mL in lovastatin-treated animals, p<0.05 by Mann-Withney test). Thus, lovastatin reduces the intracerebral and systemic inflammatory mediator responses in experimental CM. For comparison, we also treated animals with sodium diclofenac or allopurinol as control anti-inflammatory agents and did not detect protective effects in CM. In fact, sodium diclofenac increased mortality in infected animals (supplemental data 2 and 3), suggesting that unique immunomodulatory effects of lovastatin may make this drug especially suited for this condition.

### Lovastatin treatment decreases ROS production in the brains of PbA-infected mice

**Figure 7:**
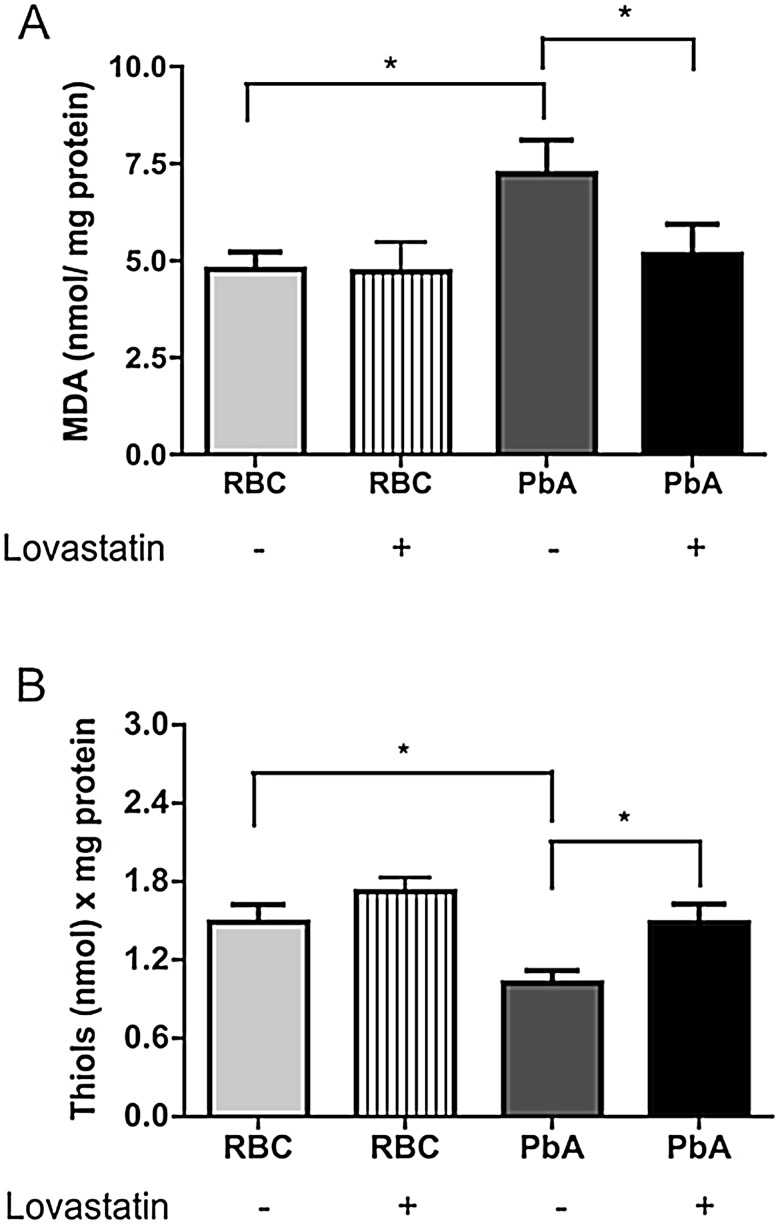
Lovastatin treatment ameliorates oxidative stress in the brains of mice with CM. Oxidative stress was assayed by measuring MDA formation (A) and free thiols levels (B) in brains 6 days post-infection of mice with PbA (10^6^ PRBC, n = 10–15/group). Control groups received the same number of uninfected RBC (10^6^). Results are expressed as mean ± S.E.M. and * represents p<0.05 compared to RBC group and # in relation to PbA-infected group, according to Tukey's Multiple Comparison Test.

We previously reported that ROS production is increased in the brain during CM and that treatment with an adjuvant combination of antioxidants prevented the cognitive impairment in animals rescued from CM by treatment with antiplasmodial drugs [9]. A similar observation was reported in a bacterial sepsis model [29]. To examine the effect of lovastatin on ROS production, we evaluated MDA and free thiol levels on day 6-post infection. As expected, MDA levels were significantly increased in the brain tissue of PbA-infected mice. This increase was abrogated by lovastatin treatment (Figure 7A). In addition, we observed evidence for significant consumption of free thiols in the brains of PbA-infected mice, which was also reversed by lovastatin treatment (Figure 7B). These results indicate that lovastatin ameliorates oxidative stress in the brains of animals with CM.

## Discussion

Neurocognitive dysfunction was detected in as many as 26% of African children surviving CM when follow-up testing was performed 6 months to 9 years after recovery from the acute disease [5], [6], [8], [30]. Considering that 500,000–800,000 children develop cerebral malaria each year in sub-Saharan Africa alone, persistent cognitive dysfunction in survivors is unquestionably a significant complication regardless of the lack of data about its occurrence in other endemic regions of the world. Importantly, the potential impact on long-term learning and behavioral function, and the socio-economical consequences in poor areas of the world where CM is a major public health concern, are likely to be extremely important but are not yet charted. It is also possible that cognitive dysfunction occurs after successful therapy of CM in adults, despite the fact that CM is much less common in older age groups [3]. Furthermore, cognitive impairment is also detected after severe bacterial sepsis – a common condition worldwide – in older patients [31], [32], and may be a frequent sequela to brain involvement in a variety of acute and severe systemic inflammatory syndromes.

The molecular mechanisms that underlie sustained cognitive dysfunction after systemic inflammatory syndromes such as CM and sepsis are not known and have been poorly investigated, making development of specific therapeutic measures equally challenging. With respect to CM, the detailed molecular mechanisms of the acute cerebral syndrome are not completely understood, although cytoadhesion of parasitized red blood cells (PRBC) and innate immune events seem to play key roles in the pathophysiology [33]. Glimpses of the molecular mechanism and mediators involved in neural dysfunctions in CM are becoming available. One analysis suggests that activation of endothelial cells in the brain vasculature and subsequent activation of microglia lead to the production of the excitotoxin quinolinic acid, a selective agonist of *N*-methyl-D-aspartate (NMDA) glutamate receptors, which can cause seizures, neuronal swelling, and delayed neuronal disintegration [34]. Pathophysiologic mechanisms of this nature may explain the neurologic complications of childhood CM.

We recently reported that the cognitive impairment is associated with the development of clinical and histological signs of CM in murine models combining plasmodium strains that incite the cerebral syndrome and mice of susceptible genetic background, and that cognitive dysfunction may be directly linked to the early inflammatory changes in the brain [9]. We found that models with systemic infection but without signs of neuroinflammation showed no cognitive impairment after cure of the parasitic disease, establishing that the cognitive dysfunction was a consequence of elements of specific brain involvement rather than severe systemic disease or the presence of the parasite *per se* [9]. We also found that blocking oxidative damage by combining anti-oxidant and antiplasmodial drugs early after the initial clinical signs of cerebral involvement was an effective combination therapy that prevented late cognitive impairment [9]. Here, we used the same permissive CM model to show that, in addition to anti-oxidants, statins are also effective adjuvant agents that prevent cognitive impairment in animals with CM. This is a novel and important observation with clear translational implications, since statins are well tolerated and widely used drugs that could easily be incorporated into clinical trials aimed at evaluating acute adjuvant therapies for patients with CM. We recognize, however, that controversy exists regarding human CM and murine models of this syndrome [3], [10]–[13], and that care must be exerted during new translational evaluation of statins in clinical trials.

If adjuvant trials of statins are undertaken, we feel that the cognitive impairment should be the main endpoint of such a study since mortality is delayed but not decreased (Figure 2B), despite clear anti-neuroinflammatory effects of statins during the acute syndrome. Supporting this approach, a recently-published study reported that another member of the statin class, atorvastatin, given therapeutically alone had no effect on survival in a model of PbA-induced CM but that a prophylactic scheme employing atorvastatin together with mefloquine, rather than mefloquine alone, yielded significant delay in mortality and onset of CM symptoms [35]. Similarly, others [36] reported that statins failed to prevent death due to CM but had an adjuvant effect when administered with artesunate, delaying death in infected animals without a clear effect on overall mortality. The delay in mortality may be secondary to a decrease in neuroinflammation and consequent death from neurological damage, leading to late mortality due to severe anemia or other complications related to the progression of the parasitic disease. We did not detect any effect of combination therapy with chloroquine and lovastatin on overall mortality, probably because the PbA strain used here is extremely sensitive to chloroquine and this drug alone was sufficient to confer a 100% rescue and survival (not shown). Furthermore, a statin (atorvastatin) was shown to synergize with mefloquine, but not with chloroquine, in *P. falciparum* killing [37]. Despite being highly effective in preventing mortality, we found that chloroquine alone did not reduce cognitive impairment in the surviving animals. This indicates that limiting the inflammatory damage to the brain, probably by shielding the endothelium against malaria-induced damage [38], was necessary to avoid the cognitive sequelae, in addition to an effective control of the parasitic disease.

In addition to their activities as cholesterol lowering drugs, statins are known to have complex pleiotropic effects, including anti-inflammatory, immunomodulatory, and endothelial function-enhancing effects both *in vitro* and in human studies [18]. Statins inhibit the mevalonate pathway, which involves the synthesis of isoprenoids and the intracellular trafficking of membrane-associated proteins such as G-proteins. GTP-binding proteins have crucial roles in intracellular inflammatory signaling by acting as molecular switches for various protein kinases. Specific targets, including Ras, Rho and Rac subfamilies, are thought to be important in systemic inflammatory syndromes, such as sepsis, because of their key roles in intracellular signaling [39]. We hypothesized that these or other molecular activities of statins prevent, or attenuate, injurious innate inflammatory responses in the brain during the initial phases of CM development. Endothelial cells are likely an important target for actions of statins that decrease CM-induced damage. In fact, atorvastatin pre-treatment of endothelial cells was recently reported to blunt the expression of adhesion molecules, reduce apoptosis, and decrease *P. falciparum* cytoadherence, while enhancing endothelial monolayer integrity during co-incubation with malaria parasites [38].

*In vitro* and animal studies have shown that statins reduce the release of TNF-α and IL-1β [18], [40]. Consistent with these observations, lovastatin decreased brain levels of IL-1β, TNF-α, IL-12 and MCP-1 in experimental CM (Figure 6). IL-1β, TNF-α, and IL-12 were previously shown to play important roles in the pathophysiology of malaria and its cerebral form [41]–[43]. There is substantial evidence that TNF-α is increased and pathogenically involved in both clinical and experimental syndromes of severe malaria [44]. Importantly, cerebrospinal fluid TNF-α levels correlated with cognitive impairment in Ugandan children [45]. In addition, elevated brain levels of IL-1β were shown to occur in children with CM and neurodegenerative lesions [46]. Furthermore, ICAM-1 expression by endothelial cells is enhanced by TNF-α and IL-1β, and ICAM-1 is important in cell adhesion, including adhesion of PRBC, in models of cerebral malaria [44], [47]. Our studies demonstrate that critical cytokines, endothelial ICAM-1, and adhesion of leukocytes and PRBC are decreased by lovastatin, and may indicate that the attenuated ICAM-1 expression detected in animals with CM treated with lovastatin is secondary to blunted cytokine signaling in these animals. These changes would be expected to have a positive impact on brain microcirculation by decreasing cell adhesion and improving blood flow, as we detected in our observations of the pial microvessels of treated animals by intravital microscopy (Figure 4). Statins also reduce inducible nitric oxide (NOS) expression, thereby reducing neuronal apoptosis, while increasing endothelial NOS expression, preserving microcirculatory blood flow via local vasodilation [48].

Blood-brain barrier disruption and edema formation are among the major features of CM [49]–[51]. Brain edema is detected in the pediatric syndrome and also in adult patients with CM studied by *in vivo* imaging techniques [52]–[54], and is of prognostic value in such patients [55]. We observed a marked increase in brain vascular permeability occurring simultaneously with detection of the initial clinical signs of CM in our experimental model. This was evidenced by quantitative measurement of Evans blue dye in brain tissue and also by histological examinations demonstrating clear evidence for perivascular edema. Previous work suggests that CM involves both cytotoxic and vasogenic edema and may, therefore, result from a combination of ischemic and inflammatory triggers [56], [57]. Vasogenic edema involves accumulation of excess fluid in the extracellular space of the brain parenchyma because of a leaky BBB [58]; cytotoxic edema consists of intracellular fluid accumulation that occurs during anoxic conditions [59]. Our findings indicate that statin administration has salutary anti-inflammatory and vascular activities in animals with CM (Figures 4, 5). Thus the inhibition of edema formation that we observed in our study may result from a combinational decrease in inflammatory damage to the BBB and obstructive plugging of microvessels. Whether cognitive dysfunction in clinical CM is directly linked to these mechanisms is yet unknown. Nevertheless, the fact that statins treatment decreases injurious neurovascular pathology and cognitive dysfunction in our model of CM suggest such a link or, at least, a common triggering mechanism.

Of relevance, expression and activation of leukocyte adhesion molecules involved in the development of endothelial damage and BBB alterations were also decreased by statins in bacterial sepsis [18], and our initial experiments indicate that statins also prevent cognitive impairment in animals recovering from bacterial sepsis (Figure 3). This observation has two major implications. First, the cognitive dysfunction detected after bacterial sepsis or CM may have common molecular pathways that are similarly affected by statins. Secondly, statins may be ideal candidates for clinical trials not only in patients with CM, but also in other patients with systemic inflammatory syndromes complicated by brain involvement. In fact, the idea that statins may be useful to reduce the burden of cognitive impairment in patients who are critically ill was suggested recently [19]. We believe that our observations are the first experimental evidence to support this possibility. Improvement of cognitive dysfunction in experimental CM and sepsis by antioxidant adjuvants also suggests that these syndromes share common molecular pathways that may be targets for similar pharmacologic therapies. Statins have also been examined in models of traumatic brain injury [60], which involves pathophysiologic changes (eg, neuronal damage and apoptosis, neuroinflammation, and BBB injury) similar to those observed in other types of critical illness, including sepsis, acute respiratory distress syndrome and CM. The benefits of statins observed in animal studies of traumatic brain injury include increased hippocampal neuron survival and improved neurologic function [61], [62]. In humans, one clinical trial reported a reduction in amnesia and increased orientation in patients with traumatic brain injury who were treated with rosuvastatin [63].

Rupture of red blood cells by *Plasmodium* parasites releases free heme, which in turn increases the expression by the host cells of the antiinflammatory enzyme HMOX-1. HMOX-1 then degrades heme to CO, iron and biliverdin [64], [65]. Increased expression of HMOX-1 after treatment with cobalt protoporphirin (CoPPIX) or CO administration prevented mortality in experimental CM [66]. These protective effects were associated with inhibition of BBB disruption, decreased brain microvascular congestion and hemorrhage, and suppression of neuroinflammation, including inhibition the expression of adhesion molecules in the brain microvasculature and the suppression of activated CD8+ T cell sequestration in the brain [66]. In addition, biliverdin, and its byproduct bilirubin, are both powerful antioxidants [67]. Previous reports have shown that statins induce HMOX-1 expression [25], [68]. In the present study we also observed that lovastatin enhanced HMOX-1 in healthy and PbA-infected mice (Figure 5I), suggesting that some of protective effects observed here result from the increase in HMOX-1 activity and the generation of its products, potentially contributing to the decrease in the inflammatory response and oxidative damage.

In conclusion, we demonstrated that a statin agent has potent salutary effects on neuroinflammation in experimental CM. Given as an adjunct therapy with antiplasmodial drugs, lovastatin prevented the cognitive impairment that follows CM. Furthermore, lovastatin treatment also prevented cognitive impairment after bacterial sepsis. We propose that statins may be valuable pharmacologic tools in treatment of patients with neuroinflammation associated with severe systemic inflammatory syndromes, and novel agents that may ameliorate cognitive dysfunction in survivors. Recognizing that translational steps from laboratory experiments to human disease must always be approached with care, our findings, together with the growing literature on statins and cognitive impairment, their safety profile, and the large experience with these drugs in patients indicate that clinical trials with statins in CM and sepsis should be considered to examine this point.

## Methods

### Animals

C57BL/6 mice (6–8 weeks old) from the FIOCRUZ breeding unit were used for the studies. The animals were kept at constant temperature (25°C) with free access to chow and water in 12-hour light/dark cycle.

### Ethics statement

The Animal Welfare Committee of the Oswaldo Cruz Foundation under license number L033/09 (CEUA/FIOCRUZ) approved the experiments in these studies. The same institution provided ethical approval created the guidelines followed by this Committee.

### Parasites and infection

Uncloned *Plasmodium berghei* ANKA (PbA) was used in this study. C57BL/6 red blood cells infected with the PbA strain were kept in liquid nitrogen and were thawed and passed into normal mice that served afterwards as parasite donors. C57BL/6 mice were inoculated intraperitoneally with 0.2 mL suspension of 10^6^ parasitized red blood cells (PRBC) (n = 10/group). As a control group for infection, mice were inoculated with 10^6^ non-parasitized red blood cells (RBC).

### Experimental design

Mice were infected as described above. On day 3 and 6, parasitemia and neurobehavioral signs of CM were monitored. CM malaria was defined by clinical evaluation of neurobehavioral parameters based on a multifactorial SHIRPA protocol as described previously [9], [69], [70]. Animals positive for CM were identified by the observation of three or more signs including: piloerection, curved trunk, alterations in gait, seizures, limb paralysis, coma, respiratory rate, skin color alterations, heart rate, lacrimation, palpebral closure, decreased grip strength, limb, abdominal and body tone and body temperature alterations. Animals that were positive for CM were immediately started on oral chloroquine treatment (25 mg/kg b.w.) [9], lovastatin (Neoquimica, Brazil, 20 mg/kg b.w., in NaCl 0.9%) [71], or both and were treated daily for 7 days. Fifteen days post-infection, animals were subjected to a battery of behavioral tests to assess cognitive function as previously described [9]. Control uninfected mice received chloroquine with or without lovastatin. On day 6 post-infection 1 h after administration of the drugs, groups of animals were examined in intravital microscopy experiments or were euthanized by isofluorane inhalation for blood and brain sampling.

### Bacterial sepsis

Feces were collected from the bowels of naïve C57BL/6 mice and macerated in 0.5 ml of sterile saline to produce a suspension at 2.5 mg/ml (w∶v). The suspension was then centrifuged (900× g–10 min). The supernatant was recovered and injected (0.5 mL, i.p) into recipient naive mice. After 6 hours, animals received 1 ml of sterile saline s.c. for fluid resuscitation and were given a broad spectrum antibiotic, imipenem (10 mg/kg b.w.), once a day for 2 consecutive days. One group of animals received lovastatin (20 mg/kg b.w., p.o.) [71] as adjuvant treatment, 1 hour before infection and concomitantly with imipenem. Control groups received the same volume of saline. The animals were followed for mortality and examined at day 15-post infection by assessment of cognitive function.

### Habituation to the open field task

Habituation to an open field was carried out as we previously described [9]. Animals were gently placed on an open field apparatus and allowed to explore the arena for 5 minutes (training session) and 24 h later they were submitted to a similar open-field session (test session). Crossing of the black lines and rearing performed in both sessions were counted.

### Step-down inhibitory avoidance test

The step-down inhibitory avoidance test was performed as we previously described [9]. In the training trial, animals were placed on a platform and their latency to step down on the grid with all four paws was measured with an automatic device. Immediately upon stepping down on the grid the animals received a 0.6 mA, 3.0-second foot shock. A retention test trial was performed 1.5 and 24 h after training and duration on the grid was recorded.

### Intravital microscopy

Mice were anesthetized with xylazine and ketamine hydrochloride (10 mg/kg b.w. and 200 mg/kg b.w., respectively, i.p.), tracheostomized, and artificially ventilated (Hugo Basile, Italy) with room air for intravital microscopy. The jugular vein was cannulated and used to inject fluorescent tracers (fluorescein isothiocyanate (FITC) bound to dextran, and rhodamine 6G). Central temperature was monitored with a rectal probe, and body temperature was maintained at 37°C with a homoeothermic blanket system (Harvard Apparatus, USA). Intravital microscopy was performed as described [72]. Animals were fixed in a stereotaxic frame, the left parietal bone was exposed by a midline skin incision, a craniotomy was performed with a high-speed drill, and the *dura mater* was incised and withdrawn to expose the cerebral pial microcirculation [73]. The cranial window was suffused with artificial cerebrospinal fluid (in mmol/L: NaCl 132, KCl 2.95, CaCl_2_ 1.71, MgCl_2_ 0.64, NaHCO_3_ 24.6, dextrose 3.71, and urea 6.7 at 37°C, pH 7.4). This procedure does not disrupt the vascular barrier of the cerebral microcirculation [74] and did not cause changes in permeability under baseline conditions. Animals were then placed under an upright fixed-stage intravital microscope with a mercury lamp (Olympus BX51/WI, USA) coupled to a CCD digital video camera system (Optronics, USA). Olympus objectives 4×, 10× and 20× were used in the experiments and produced a total magnification of 40×, 100× and 200×, respectively.

### Capillary density and evaluation of leukocyte-endothelial interactions

After intravenous administration of 0.1 ml of 5% FITC-labeled dextran, microscopic images of the cerebral microcirculation were acquired by Archimed 3.7.0 software and capillaries were counted by Saisam software (Microvision, France). Functional capillary density, considered as the total number of spontaneously perfused capillaries (vessels with diameters less than 10 µm) per square millimeters of surface area (1 mm^2^), was determined in random microscopic fields during 4 minutes [75].

Circulating leukocytes were labeled by administration of rhodamine 6G (0.3 mg/kg b.w., i.v.) and fluorescence associated with leukocytes was visualized by epi-illumination at 510 to 560 nm, using a 590 nm emission filter. Five randomly selected venular segments (30–100 µm in diameter) were observed for 30 seconds in each preparation for leukocyte recruitment. The leukocyte-endothelial interactions were evaluated by determining the number of leukocytes adherent to 100 µm of the venular wall for a period of 30 seconds. Rolling leukocytes were defined as crossing the venular segment at a speed below the circulating red blood cells and expressed as number of cells/min [74].

### Immunohistochemistry

After perfusion with 4% buffered paraformaldehyde, brains were fixed for 6 h, cryoprotected with 30% sucrose in PBS and sectioned on a freezing microtome. Free-floating sections (40 mm) were treated to eliminate endogenous peroxidase activity (20% methanol containing 1% H_2_O_2_) for 30 min and nonspecific binding in blocking solution (20% normal goat serum, 20% bovine serum albumin in Tris-buffered saline containing 0.1% Triton X-100) for 1 h at room temperature. Antibodies were diluted in blocking solution. Hamster anti-mouse monoclonal antibody against ICAM-1 (BD Pharmigen) was diluted 1∶200 and permitted to bind overnight at 4°C. Biotinylated hamster antibody (Vector Laboratories, Burlingame, CA, USA) was then applied at a dilution of 1∶1000 for 1 h at room temperature. Antibody binding was visualized using Vectastain ABC kit (Vector Laboratories) and diaminobenzidine as chromogen according to the manufacturer and counterstained with haematoxylin-eosin.

### Evaluation of blood brain barrier integrity

Mice were injected (i.v.) with 0.2 ml of 2% Evans blue dye (Sigma) on day 6 and 7-post infection 1 hour after the treatment with lovastatin and were sacrificed 1 h later. The brain was weighed and placed in formamide (3 ml, 37°C, 48 h) to extract Evans blue dye from the tissue. Absorbance was measured at 620 nm. Evans blue dye concentration was calculated by using a standard curve. Data are expressed as oedema inhibition index calculated as [100-(PbA-Treated mice oedema values×100/PbA oedema values)].

### Reverse transcription-polymerase chain reaction (RT-PCR) analysis

Total RNA was prepared from brain samples from mice using Trizol reagent (Life Technologies, CA, USA) according to the manufacturer's instructions. Total RNA was reverse-transcribed by using the Superscript III First-Strand Synthesis for RT-PCR kit (Life Technologies, CA, USA) with 1 µg total RNA and oligo dT. Primer sequences were as follows: ICAM-1, sense: 5′-agcttgcacgacccttctaa-3′, antisense: 5′-agcacctccccacctacttt-3′; CD11b, sense: 5′-tgaatggggacaaactgca-3′, antisense: 5′-gccatccattgtgagatcct-3′; HMOX-1, sense: 5′- ccagagtgttcattcgagca-3′, antisense: 5-cacgcatatacccgctacct-3′; GAPDH, sense: 5′- ccaggttgtctcctgcgact-3′, antisense: 5′-ataccaggaaatgagcttgacaaagt-3′. DNA sequences were chosen using the NCBI/Nucleotide bank and then converted using the Primer 3 program [76]. PCR amplification of the resulting cDNA template was conducted by using the following conditions for 35 cycles. After an initial denaturation step at 95°C for 15 min, temperature cycling was initiated. Each cycle consisted of denaturation at 94°C for 30 sec, annealing at 60°C for 30 sec, and elongation at 72°C for 30 sec. PCR products were analyzed on 6% polyacrylamide gel and stained by silver dying. Images were captured and analyzed by Image J software (National Institute of Health, USA).

### Cytokine and chemokine production during CM

Brain tissue and serum were frozen for cytokine measurements (IL-β, IL-12, and TNF-α) by enzyme-linked immunosorbent assay (ELISA, Duo set kit - R&D systems, Minneapolis, MN, USA) accordantly to the manufacturer's instructions. Brains were homogenized in phosphate-buffered saline containing 2% sodium dodecyl sulfate with 50 mM protease inhibitor cocktail (Roche, AG, Basel, Switzerland) and then centrifuged (12,000× g at 4°C for 20 min). The supernatant was collected and protein content was evaluated by the Bicinchoninic Acid (BCA) Assay [77].

### Assessment of oxidative stress

To characterize the oxidative stress in murine brains we measured thiobarbituric acid reactive species – TBARS [77]- and the consumption of free thiols [78]. Brains were homogenized in cold phosphate buffer, pH 7.4 with BHT (2,6-di-tert-butyl-4-methylphenol) 0.2%. Briefly, the samples (0.5 mL) were mixed with equal volume of thiobarbituric acid 0.67% (Sigma Chemical, USA) and then heated at 96°C for 30 min. TBARS were determined by the absorbance at 535 nm. To analyze free thiol levels, samples were precipitated with TCA 10%, centrifuged, and the supernatant was added to DTNB (5,5′′-Dithiobis-2-nitrobenzoic acid - 1,7 M) and Tris-HCl buffer (30 mM, containing Ethylenediamine tetra-acetic acid-EDTA 3 mM, pH 8.9). Results were expressed as malondialdehyde (MDA, ε = 1.56×10^5^M^−1^cm^−1^) and thiol (ε = 1.415×10^4^M^−1^cm^−1^) in nmol/mg of protein.

### Statistical analysis

Data were expressed as mean ± SEM. Data from the open-field and inhibitory avoidance tasks were analyzed with ANOVA followed by Tukey's post-hoc (training session) and Student's T or Mann–Whitney U tests (training and test comparison) and expressed as mean ± SEM. Statistical significance of survival curves was evaluated by Log-rank (Mantel-Cox) and Gehan-Breslow-Wilcoxon tests. Data from intravital microscopy were analyzed by ANOVA with Bonferroni test and reported as means ± SEM. Differences in amounts of ICAM-1, CD11b and HMOX-1 relative expression, cytokine/chemokine, MDA, and thiol levels were evaluated by Student's t or Mann–Whitney U tests. P values of 0.05 or less were taken as statistical significance.

## Supporting Information

Figure S1 Lovastatin treatment reduces pro-inflammatory MCP-1levels in the brains of animals with CM MCP-1 (A), and RANTES (B) levels were determined by ELISA in brains of mice 6 days after infection with PbA (10^6^ PRBC, n = 6–10/group). *p<0.05 or less (Tukey's Multiple Comparison test) in relation to non-infected group.
(TIFF)

Figure S2 Sodium diclofenac increases lethality in mice treated with chloroquine. Mice were infected with PbA and were treated with chloroquine (25 mg/kg) once a day for 7 days at the first signs of CM (day 6 post-infection) or with chloroquine+sodium diclofenac (50 mg/kg b.w. p.o.) administered together with chloroquine. On day 12 we observed 100% of mortality in animals treated with chloroquine+sodium diclofenac. P<0.05 when compared with chloroquine treated mice (Log-rank (Mantel-Cox) and Gehan-Breslow-Wilcoxon tests).
(TIFF)

Figure S3 Allopurinol did not prevent cognitive impairment after cerebral malaria. C57BL/6 mice (n = 12–20/group) were infected with PbA (10^6^ PRBC). As a control, one group was inoculated with the same number of uninfected RBC (n = 6–12/group). Starting on day 6-post infection, uninfected and PbA-infected mice were divided into 2 groups and treated orally with chloroquine (25 mg/kg b.w.), or with the combination of chloroquine+allopurinol (100 mg/kg b.w. p.o.) for 7 days. On days 15 and 16 post-infection all the animals were submitted to open field training and test session (A–B). Data are expressed as mean ± S.E.M. of crossings (A) and rearings (B) in training (*gray bars*) and test (*black bars*) sessions. *Significant difference between groups in training and test sessions (*p*<0.05, Student's t test).
(TIFF)

Video S1 Venules with adherent and rolling rhodamine-labeled leukocytes in uninfected mice treated with vehicle.
(MPG)

Video S2 Venules with adherent and rolling rhodamine-labeled leukocytes in uninfected mice treated with lovastatin (20 mg/kg b.w.).
(MPG)

Video S3 Venules with adherent and rolling rhodamine-labeled leukocytes in PbA-infected mice treated with vehicle.
(MPG)

Video S4 Venules with adherent and rolling rhodamine-labeled leukocytes in PbA-infected mice treated with lovastatin (20 mg/kg b.w.).
(MPG)
