# Supplementary figures and images for: Statins Decrease Neuroinflammation and Prevent Cognitive Impairment after Cerebral Malaria
Source: PLoS Pathog. 2012 Dec 27;8(12):e1003099. doi: 10.1371/journal.ppat.1003099 (PMC3531520; doi:10.1371/journal.ppat.1003099)

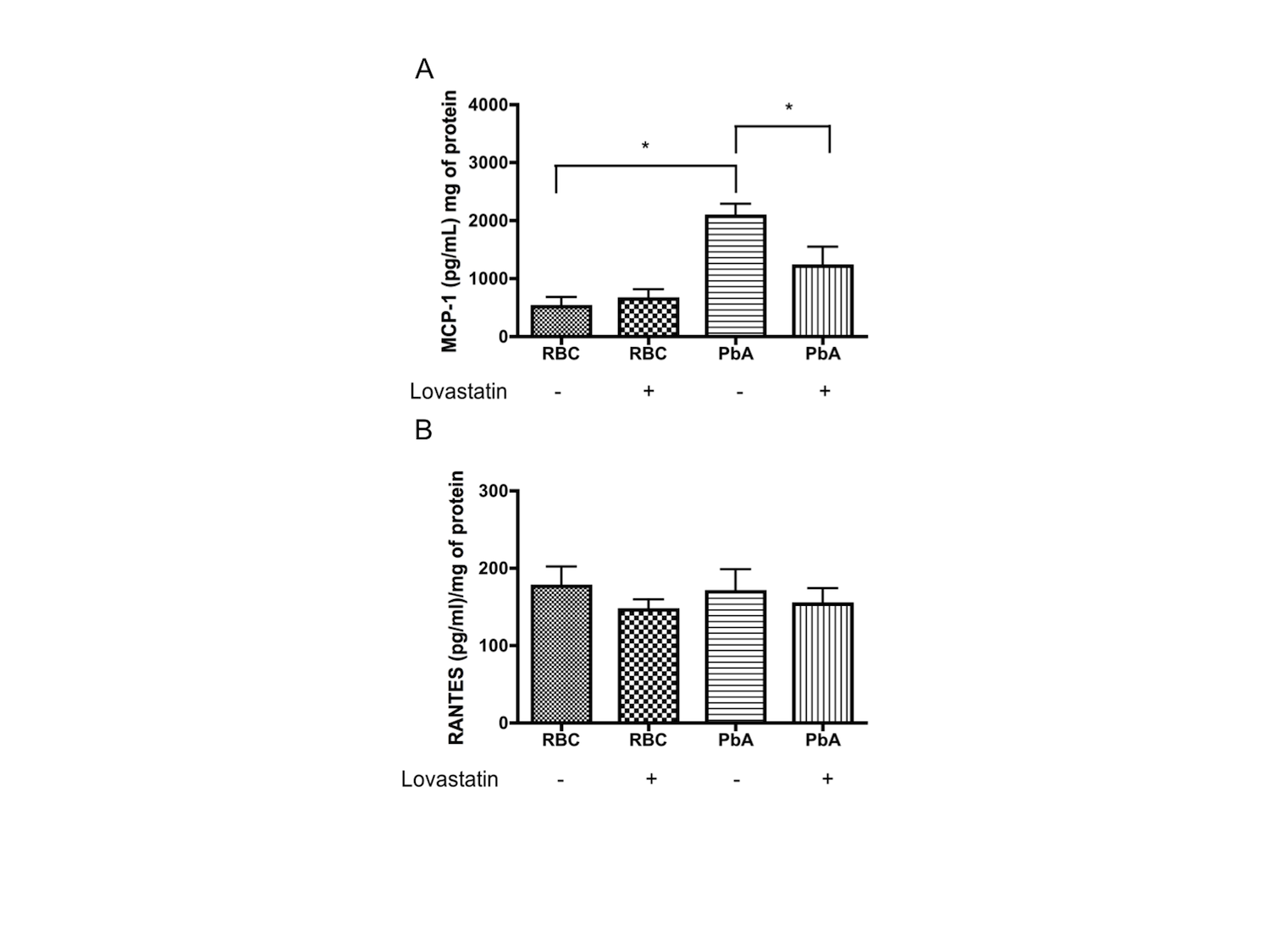

Supplement: Figure S1 — Lovastatin treatment reduces pro-inflammatory MCP-1levels in the brains of animals with CM MCP-1 (A), and RANTES (B) levels were determined by ELISA in brains of mice 6 days after infection with PbA (106 PRBC, n = 6–10/group). *p<0.05 or less (Tukey's Multiple Comparison test) in relation to non-infected group. (TIFF) [file ppat.1003099.s001.tiff]

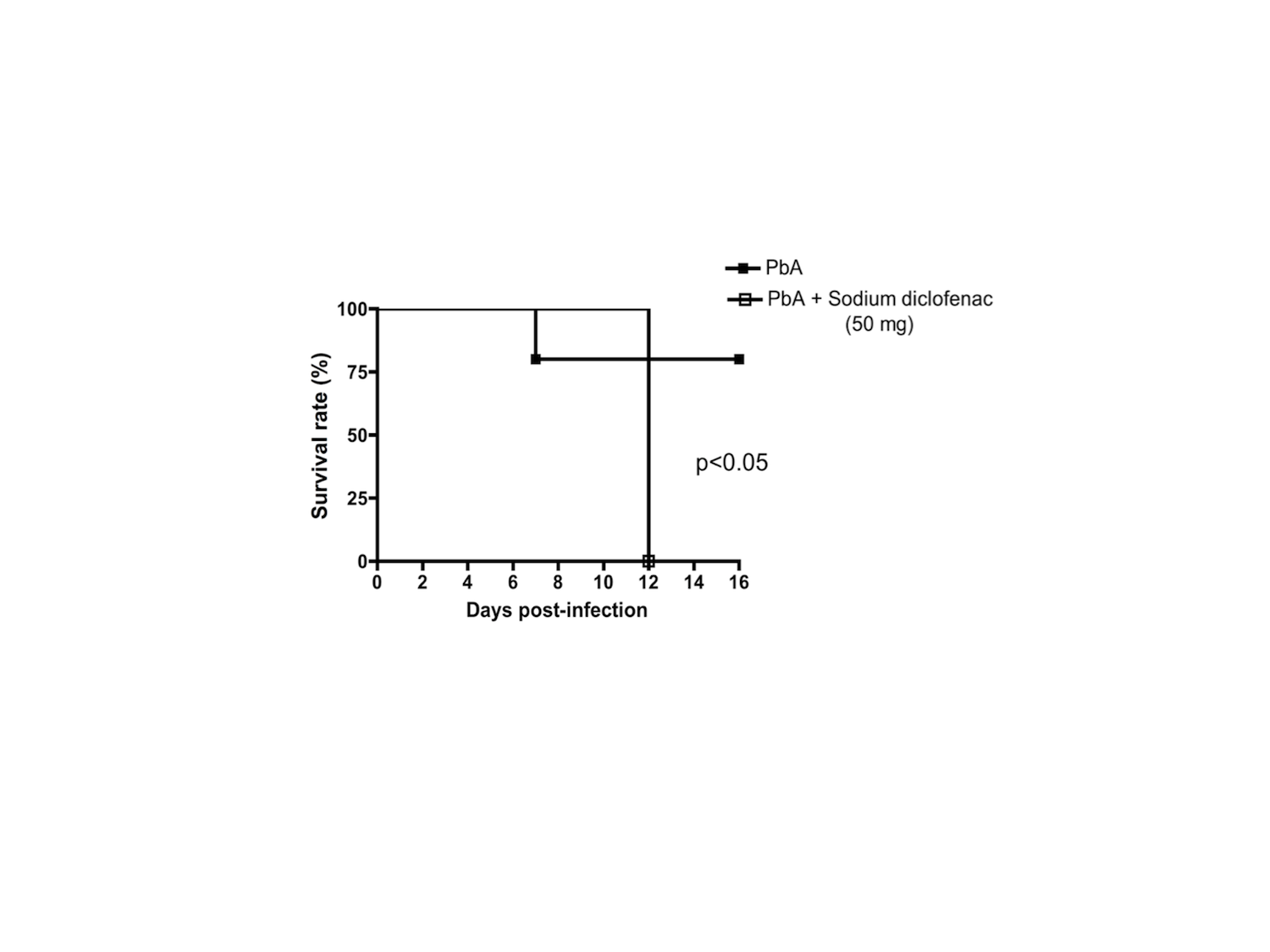

Supplement: Figure S2 — Sodium diclofenac increases lethality in mice treated with chloroquine. Mice were infected with PbA and were treated with chloroquine (25 mg/kg) once a day for 7 days at the first signs of CM (day 6 post-infection) or with chloroquine+sodium diclofenac (50 mg/kg b.w. p.o.) administered together with chloroquine. On day 12 we observed 100% of mortality in animals treated with chloroquine+sodium diclofenac. P<0.05 when compared with chloroquine treated mice (Log-rank (Mantel-Cox) and Gehan-Breslow-Wilcoxon tests). (TIFF) [file ppat.1003099.s002.tiff]

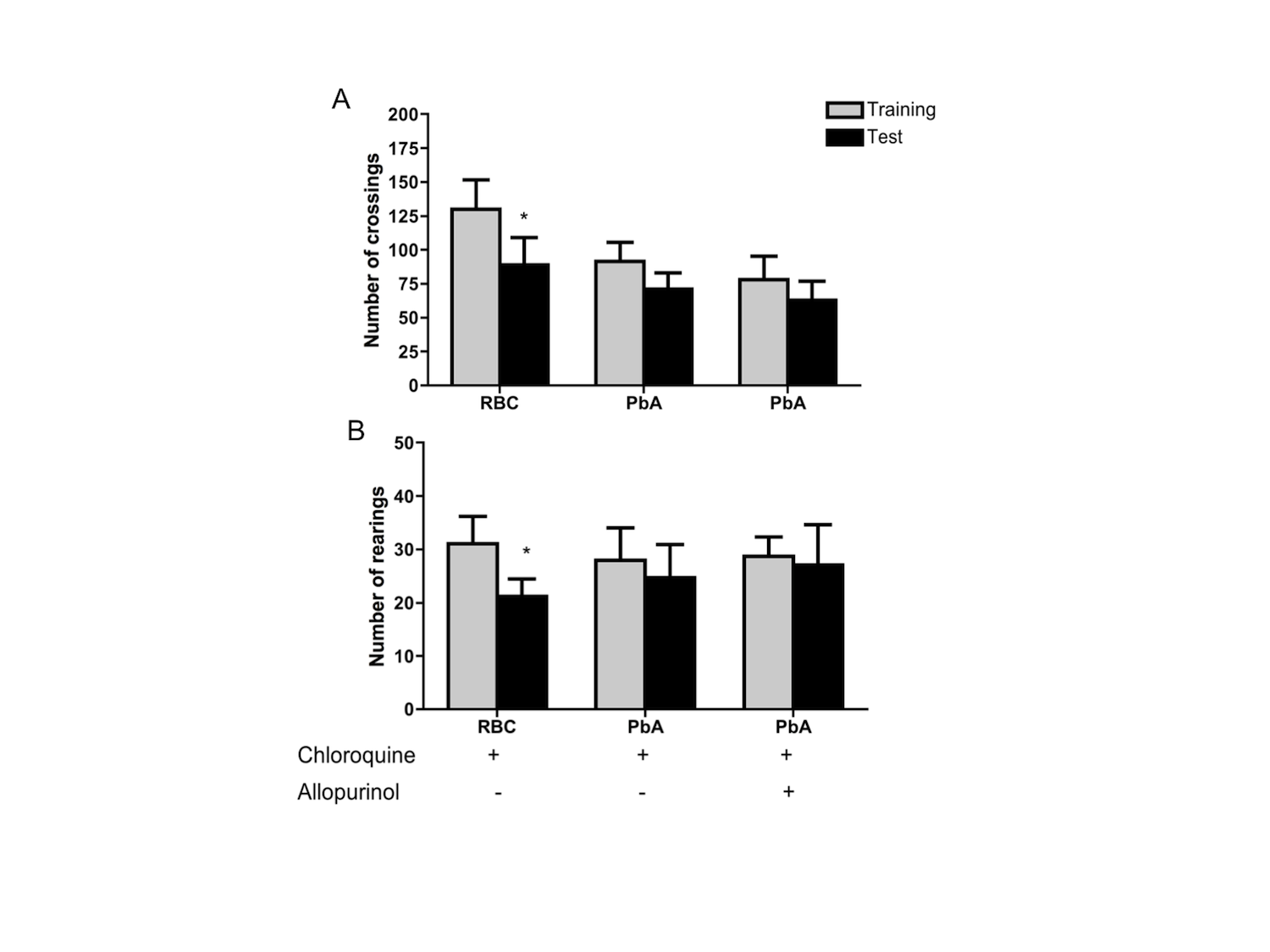

Supplement: Figure S3 — Allopurinol did not prevent cognitive impairment after cerebral malaria. C57BL/6 mice (n = 12–20/group) were infected with PbA (106 PRBC). As a control, one group was inoculated with the same number of uninfected RBC (n = 6–12/group). Starting on day 6-post infection, uninfected and PbA-infected mice were divided into 2 groups and treated orally with chloroquine (25 mg/kg b.w.), or with the combination of chloroquine+allopurinol (100 mg/kg b.w. p.o.) for 7 days. On days 15 and 16 post-infection all the animals were submitted to open field training and test session (A–B). Data are expressed as mean ± S.E.M. of crossings (A) and rearings (B) in training (gray bars) and test (black bars) sessions. *Significant difference between groups in training and test sessions (p<0.05, Student's t test). (TIFF) [file ppat.1003099.s003.tiff]
